# Multi-Size Deep Learning Based Preoperative Computed Tomography Signature for Prognosis Prediction of Colorectal Cancer

**DOI:** 10.3389/fgene.2022.880093

**Published:** 2022-05-12

**Authors:** Cheng-Hang Li, Du Cai, Min-Er Zhong, Min-Yi Lv, Ze-Ping Huang, Qiqi Zhu, Chuling Hu, Haoning Qi, Xiaojian Wu, Feng Gao

**Affiliations:** ^1^ Department of Colorectal Surgery, The Sixth Affiliated Hospital, Sun Yat-sen University, Guangzhou, China; ^2^ Guangdong Institute of Gastroenterology, Guangdong Provincial Key Laboratory of Colorectal and Pelvic Floor Disease, Supported by National Key Clinical Discipline, The Sixth Affiliated Hospital, Sun Yat-sen University, Guangzhou, China; ^3^ School of Computer Science and Engineering, Guangzhou Higher Education Mega Center, Sun Yat-sen University, Guangzhou, China; ^4^ Ningbo Medical Center Lihuili Hospital, Ningbo, China

**Keywords:** deep learning, colorectal cancer, prognosis, nomogram, pathway analysis

## Abstract

**Background:** Preoperative and postoperative evaluation of colorectal cancer (CRC) patients is crucial for subsequent treatment guidance. Our study aims to provide a timely and rapid assessment of the prognosis of CRC patients with deep learning according to non-invasive preoperative computed tomography (CT) and explore the underlying biological explanations.

**Methods:** A total of 808 CRC patients with preoperative CT (development cohort: *n* = 426, validation cohort: *n* = 382) were enrolled in our study. We proposed a novel end-to-end Multi-Size Convolutional Neural Network (MSCNN) to predict the risk of CRC recurrence with CT images (CT signature). The prognostic performance of CT signature was evaluated by Kaplan-Meier curve. An integrated nomogram was constructed to improve the clinical utility of CT signature by combining with other clinicopathologic factors. Further visualization and correlation analysis for CT deep features with paired gene expression profiles were performed to reveal the molecular characteristics of CRC tumors learned by MSCNN in radiographic imaging.

**Results:** The Kaplan-Meier analysis showed that CT signature was a significant prognostic factor for CRC disease-free survival (DFS) prediction [development cohort: hazard ratio (HR): 50.7, 95% CI: 28.4–90.6, *p* < 0.001; validation cohort: HR: 2.04, 95% CI: 1.44–2.89, *p* < 0.001]. Multivariable analysis confirmed the independence prognostic value of CT signature (development cohort: HR: 30.7, 95% CI: 19.8–69.3, *p* < 0.001; validation cohort: HR: 1.83, 95% CI: 1.19–2.83, *p* = 0.006). Dimension reduction and visualization of CT deep features demonstrated a high correlation with the prognosis of CRC patients. Functional pathway analysis further indicated that CRC patients with high CT signature presented down-regulation of several immunology pathways. Correlation analysis found that CT deep features were mainly associated with activation of metabolic and proliferative pathways.

**Conclusions:** Our deep learning based preoperative CT signature can effectively predict prognosis of CRC patients. Integration analysis of multi-omic data revealed that some molecular characteristics of CRC tumor can be captured by deep learning in CT images.

## Introduction

Colorectal cancer (CRC) is one of the most prevalent cancers and has become the third leading cause of cancer death (Siegel et al., 2020). Stratification of CRC patients is quite essential to design more accurate and personalized treatment according to their clinical characteristics (Sorbye et al., 2007). Though the current tumor-node-metastasis (TNM) system has been used for guiding treatment decisions of CRC patients for over 50 years (Nagtegaal et al., 2011), it is still inadequate for accurately assessing the prognosis of some colorectal patients, especially for patients in clinical stage II and III (Joachim et al., 2019). Even with the same clinical stage, patients may be suitable to different treatment options before and after surgery as heterogeneity of CRC (Molinari et al., 2018). Thus, prognostic analysis of CRC patients and evaluation of their preoperative and postoperative interventional treatment options are recent research hotspots.

Previous studies on the molecular basis of cancer and the discovery of cancer associated genes, oncogenes and tumor suppressor genes indicates that cancer is a genetic disease (Pierotti, 2017), which determines a natural advantage for cancer survival analysis with genomics data (Walther et al., 2009; Yu et al., 2015). However, the expensive cost and long detection time severely limit its mass adoption. Radiomics is a high-throughput analysis of quantitative tumor characteristics from standard-of-care medical imaging, like computed tomography (CT) and magnetic resonance imaging (MRI). By further modeling with machine learning, radiomics can provide better clinical-decision support systems for the clinicians, like tumor diagnosis and prognosis prediction (Lambin et al., 2017). Compared with genetic detection, radiographic testing is non-invasive and does little harm to the weak patients. Especially, comparing with MRI, CT is much cheaper, and its examination results can be available faster. As a preoperative routine test for CRC patients to locate the tumor before resection surgery, CT imaging analysis can provide timely guidance on surgical procedures and postoperative treatment. With sophisticated image processing tools to obtain high-dimensional image features, CT images contain abundant information which provides a powerful application in multiple medical studies (Limkin et al., 2017).

Typical radiomic features are mainly morphological characteristics of the tumor lesion, such as tumor size, shape and texture, which are customized according to human recognition cognition or compliant with certain human-defined rules (Gillies et al., 2016). The standardization of radiomic features makes it possible to quantify phenotypic characteristics on medical imaging (van Griethuysen et al., 2017). Through successfully applications in tumor differentiated grading (Kim et al., 2015), genomics prediction (Yang et al., 2018), prognosis predicting (Huang et al., 2016), evaluation of tumor immune microenvironment (Jiang et al., 2020) and prediction of chemoradiation therapy response (Shi et al., 2019), radiomic studies demonstrate that radiographic images can provide abundant information for cancer research. However, these radiomics features obtained by typical method are still limited by the human definition. It fails to consider the feature-to-feature relationship which plays a vital role in tumor microenvironment. Deep learning, one kind of machine learning based on artificial neural networks, has a powerful ability in image analysis (LeCun et al., 2015) with convolutional neural networks (CNNs). A few studies based on deep learning have proved its effectiveness in tumor assessment like lymph node status prediction (Zheng et al., 2020) and tumor recurrence prediction (Liu et al., 2022). Though with high prediction accuracy, deep learning is known as a black box as lacks the interpretation for its prediction, which makes it hard to be accepted by doctors. Excavating the hidden biological mechanism for the deep learning models will improve its interpretability and promote the clinical utility. Thus, there is an urgent need for developing a biologically interpretable deep learning model for predicting the prognosis of colorectal cancer.

In this study, we investigated an end-to-end CNNs model to quantify radiographic tumor characteristics and prognosis prediction for CRC patients. Deep features of CT images were extracted for correlation analysis with RNS-seq data from the ICGC-ARGO project (The International Cancer Genome Consortium-Accelerating Research in Genomic Oncology) to further explore the underlying biological mechanism learned by the Multi-Size Convolutional Neural Network (MSCNN) model. Our results proved that deep CT features can reveal the molecular information of tumors to some extent and ultimately improve the stratification of CRC patients.

## Materials and Methods

### Patients and Data Collection

In this retrospective study, a total of 808 colorectal cancer patients who had cancer resection at the Sixth Affiliated Hospital of Sun Yat-sen University from 22 Jan 2008 to 30 Jan 2018 were included for analysis. Patients admitted during 2008-2013 were assigned to the development cohort (*n* = 426) for model construction and the rest of patients admitted during 2014-2018 were assigned to the validation cohort (*n* = 382) for model validation. All patients had CT examinations before the cancer resection surgery and the image data were stored in DICOM (Digital Imaging and Communications in Medicine) format. Region of interest (ROI) for colorectal cancer tumor area was manually delineated by experienced doctors with ITK-snap (Version 3.2) software. Baseline clinicopathological information containing age, gender, differentiated grade, lymph node metastasis and microsatellite status. Among these patients, 236 patients were enrolled in the ICGC-ARGO project and had paired RNA sequencing data.

### Data Preprocessing and Enhancement

**FIGURE 1:**
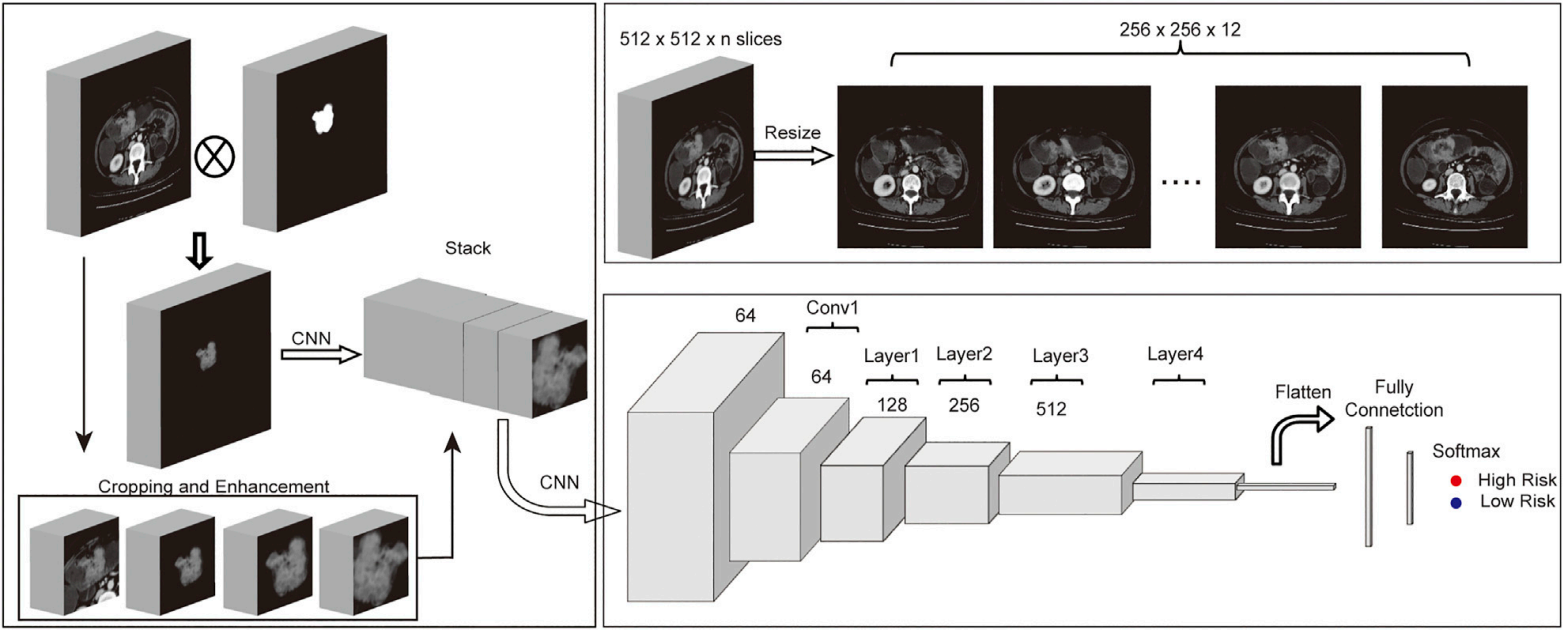
Workflow of MSCNN. **(A)** Multi-Size based data enhancement of CT images before fed into MSCNN. **(B)** Data preprocessing of CT images with ROIs. **(C)** Network structure of MSCNN Multi-Size which includes a CNN to combine Multi-Size CT data, a ResNet34 network to extract image features of tumors from CT images and a last classification network.

Figure 1 shows the pipeline of our analysis from origin CT images and their corresponding ROI to predict the disease-free survival for each patient. Origin CT images size is 512 * 512 with slices from 23 to 682 (mean = 162), and the valid slices which have tumor lesion of ROI for each CT image range between 3 and 77. To fit the deep learning model and reduce the computational parameters, all 3D CT images and ROIs only kept the slices with valid areas and then were resized to 256 * 256 * 12 with SciPy ndimage python submodule. To better conclude the tumor boundary information, all ROIs were binarily dilated with five pixels using morphology function in ndimage submodule. As the tumor ROI area of colorectal cancer is usually quite small, accounting for only 1–5% of the whole CT image, detailed information for the tumor is hard to extract from the deep learning model. To address this issue, the tumor area is cropped and magnified at different magnifications. Meanwhile, the cropped CT images were also augmented by rotating at random angles and flipping with a certain probability. Finally, all images for each patient were stacked together to feed into the neural network.

### Multi-Size Convolutional Neural Network Model Construction

Convolutional Neural Network (CNN) is a powerful Deep Learning algorithm that can extract relevant texture features from the image. By stacking several CNNs, deep learning model can learn deeper features from the image according to the training task. Although model becomes much difficult to train if there are too many layers in deep learning networks, a residual neural network (ResNet (He et al., 2016)) is designed to solve this problem. Our model was based on ResNet34, which contains 34 convolutional neural networks and four residual blocks. First, one subnetwork with CNNs of different input sizes were designed for features extraction from the origin CT image and its enhanced cropped images. Then all features from these CNN were stacked together and following one CNN layer and the rest residual blocks of RenNet34 were used to extract higher and deeper features. Finally, one Fully Connected (FC) layer which contains one hidden layer with 64 nodes and one output layer finished the patient disease-free survival classification task.

### Model Development and Validation

As shown in Supplementary Figure S1, for better training the model, only patients with tumor recurrence in 3 years or disease-free survival for more than 5 years were considered in the model development stage. CT images with ROI were fed into the deep learning model, and disease-free survival status was used as the labels. Model training was performed by updating the network weights using the backpropagation algorithm according to the cross-entropy loss between the prediction and the real outcomes. Adam optimizer was used in model network weights updating, and the learning rate was decayed to half for every 10 training epochs with an initial rate of 0.001. During training, the loss was continuously monitored, and model weights were saved when loss decreased. If the loss was not decreased for more than 20 epochs, then training was ended and saved model with the highest Area under the receiver operating characteristic (ROC) Curve (AUC) was loaded for further validation. CT signatures score was calculated on the whole development and validation cohort through the MSCNN model with CT images. A nomogram was constructed by incorporating the CT signature with other clinicopathologic risk factors, and its benefit was evaluated by the calibration curve and Decision curves analysis (DCA).

### Radiomics Method

To compare our deep learning based method with conventional radiomics method, we constructed a model with CT radiomics features. For each of CT image, a total of 107 radiomics features were extracted using Pyradiomics (van Griethuysen et al., 2017) package in python 3.8 platform. Standard Deviation (SD) and Median Absolute Deviation (MAD) were used to initially screen features with significant differences. Z-score normalization was performed to increase the comparability between the left radiomics features. The least absolute shrinkage and selection operator (LASSO) with cox regression was used to construct the final radiomics based model.

### Deep Features Visualization

To visualize how the MSCNN divides patients into high recurrence risk and low recurrence risk, deep features from the last two layers of the MSCNN model were exported for further analysis. A correlation heatmap was performed on the 64 features from the hidden notes of the FC layer to show the most related deep CT features with high recurrence risk and low recurrence risk. Principal component analysis (PCA) analysis was performed on 512 origin deep CT features from the ResNet34 network and 64 features from hidden nodes of the FC layer.

### Correlating the Computed Tomography Signature and Deep Computed Tomography Features With Gene Expression Data

To explore the biological characteristics of CT signature, Gene Ontology analysis and Gene Set Enrichment Analysis (GSEA) was conducted for differentially expressed genes between the risk groups. To further figure out how the model captures the underlying biological information from CT images, correlation analysis was performed between 64 deep CT features and cancer-related pathways. Functional spectra were calculated with the DeepCC method to explore the most related biological pathways with deep CT features (Gao et al., 2019). All hallmark pathways which have significant correlations with these 64 deep CT features were displayed in a bar plot.

### Statistical Analyses

All statistical analyses were performed by R software (version 4.1.1). Kaplan-Meier curve was used to perform survival analysis for model prediction results with R package “survival”. Log-rank test was used to evaluate results of the univariable analysis of model prediction results and other clinic-pathological factors with disease-free survival (DFS). Multivariable analysis was performed using the Cox proportional hazards regression method with only the significant variables from univariable analysis. Correlation analysis were performed using the Pearson method. For all analyses, the two-sided value *p* value < 0.05 was considered statistically significant.

## Results

### Risk Prediction From Computed Tomography Images

We calculated the recurrence risk of colorectal cancer patients with CT images and ROI in an end-to-end deep learning method. After model training with the development cohort, a CT signature score of each patient was calculated with the MSCNN model in Supplementary Table S1. Patients with a recurrence risk of more than 0.5 were classified into high risk groups, and the remain patients were in low risk group. Patients’ clinical characteristics in development and validation cohort were displayed in Table 1.

**TABLE 1:** Baseline characteristic of patients in the development and validation cohort.

	level	Development cohort(*n* = 426)			Validation cohort (*n* = 328)		
		Low Risk	High Risk	P	Low Risk	High Risk	P
n		268	158		200	182	
Age (mean (SD))		58.732 (12.676)	59.816 (15.649)	0.4878	56.799 (13.099)	57.134 (13.191)	0.833
Sex (%)	F	116 (43.28)	57 (36.08)	0.1735	92 (46.00)	68 (37.36)	0.1085
	M	152 (56.72)	101 (63.92)		108 (54.00)	114 (62.64)	
TNM stage (%)	I	28 (10.45)	7 (4.43)	<0.0001	38 (19.19)	35 (19.34)	0.0026
	II	126 (47.01)	29 (18.35)		69 (34.85)	42 (23.20)	
	III	99 (36.94)	53 (33.54)		63 (31.82)	52 (28.73)	
	IV	15 (5.60)	69 (43.67)		28 (14.14)	52 (28.73)	
T stage (%)	T1	14 (5.22)	5 (3.18)	0.0001	9 (4.55)	8 (4.42)	0.6512
	T2	23 (8.58)	4 (2.55)		35 (17.68)	29 (16.02)	
	T3	208 (77.61)	112 (71.34)		133 (67.17)	117 (64.64)	
	T4	23 (8.58)	36 (22.93)		21 (10.61)	27 (14.92)	
N stage (%)	N0	157 (58.80)	51 (32.90)	<0.0001	118 (59.00)	84 (46.15)	0.0175
	N1	84 (31.46)	65 (41.94)		58 (29.00)	60 (32.97)	
	N2	26 (9.74)	39 (25.16)		24 (12.00)	38 (20.88)	
M stage (%)	M0	253 (94.40)	89 (56.33)	<0.0001	172 (88.21)	130 (78.31)	0.0168
	M1	15 (5.60)	69 (43.67)		23 (11.79)	36 (21.69)	
Differentiation grade (%)	Low	57 (30.81)	29 (26.36)	0.0256	45 (36.00)	29 (25.44)	0.181
	Moderate	117 (63.24)	64 (58.18)		76 (60.80)	79 (69.30)	
	High	11 (5.95)	17 (15.45)		4 (3.20)	6 (5.26)	
Chemotherapy Adjuvant (%)	No	68 (32.69)	32 (32.65)	1	108 (56.84)	102 (61.82)	0.3992
	Yes	140 (67.31)	66 (67.35)		82 (43.16)	63 (38.18)	

### High Risk and Low Risk Patients Show Significant Different Survival

**FIGURE 2:**
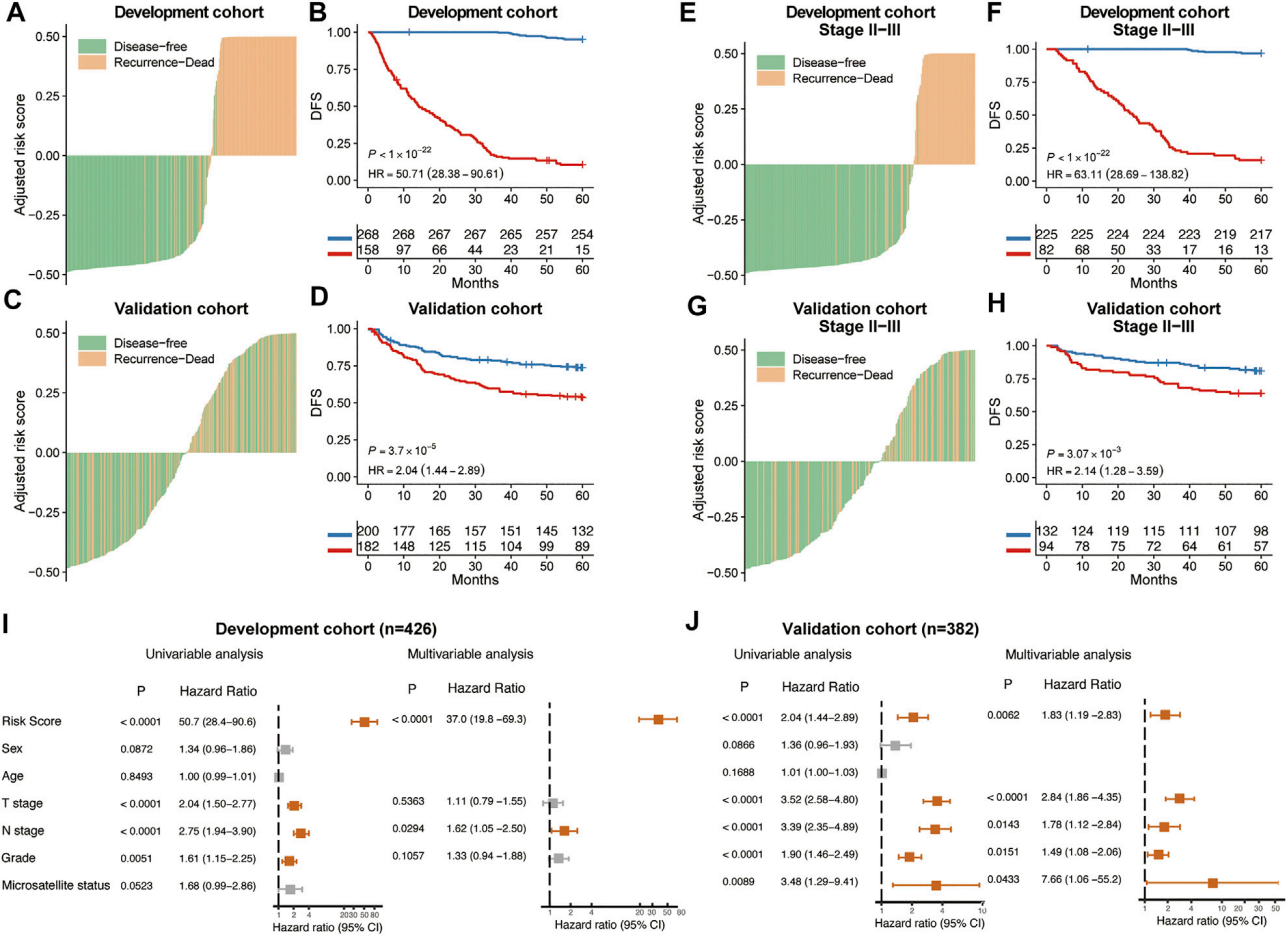
Prognostic performance of MSCNN. The distribution of CT signature of MSCNN and its corresponding recurrence status in the development cohort **(A)** and validation cohort **(C)**. Kaplan-Meier curves showed a significant survival difference between the high and low risk groups in the development cohort **(B)** and validation cohort **(D)**. Prognostic analysis of CRC patients in stage II and III subgroups **(E–H)**. Univariable and multivariable analysis of clinical factors in the development cohort **(I)** and validation cohort **(J)**.

In both development and validation cohorts, high risk patients show worse mean survival (23 vs. 105 months and 46 vs. 58 months). Kaplan-Meier curve revealed a significant association between CT images risk prediction and patients’ DFS in the development cohort (HR: 50.7, 95% CI: 28.4–90.6, *p* < 0.001) and validation cohort (HR: 2.04, 95% CI: 1.44–2.89, *p* < 0.001) (Figures 2A–D). Previous research showed that clinicopathological information may be not enough to accurately predict the recurrence risk for colorectal patients with stage II and III (Tsikitis et al., 2014). Kaplan-Meier survival curve in stage II and III patients showed that risk prediction of our model can still divide those patients into significant survival different groups (Figures 2E–H). Univariable and multivariable cox regression analyses were performed to identity significant clinicopathological factors associated with cancer recurrence. Besides the risk scores calculated from CT images, clinical factors sex, age, T stage, N stage, differentiation grade and Microsatellite status were added to multivariable analysis. Forest plot showed risk scores from CT images was an independent prognostic predictor of cancer recurrence in both development and validation cohorts (Figures 2I,J).

### Radiomics Model and Risk Prediction

Standard Deviation and Median Absolute Deviation for each radiomics features was calculated after z-score normalization and only 50 features with SD > 1 and MAD > 3.5 were left for subsequent modeling. Finally, 11 radiomics features were kept with LASSO-cox regression to construct the classification model. Radiomics score was calculated by a linear combination of non-zero coefficients multiplied with these 11 radiomics features. To classify high and low risk groups, the optimal cut-off of radiomics scores was determined by the time-dependent ROC curve. Survival analysis showed significant differences between high risk patients and low risk patients according to radiomics scores (Supplementary Figures S2B,D). Comparison between our MSCNN method and Radiomics method were displayed with ROC curves and the result proved that our model could obtain better prediction of prognosis in both development and validation cohorts (Supplementary Figures S2A,C).

### Nomogram for Risk Prediction From Radiomics

**FIGURE 3:**
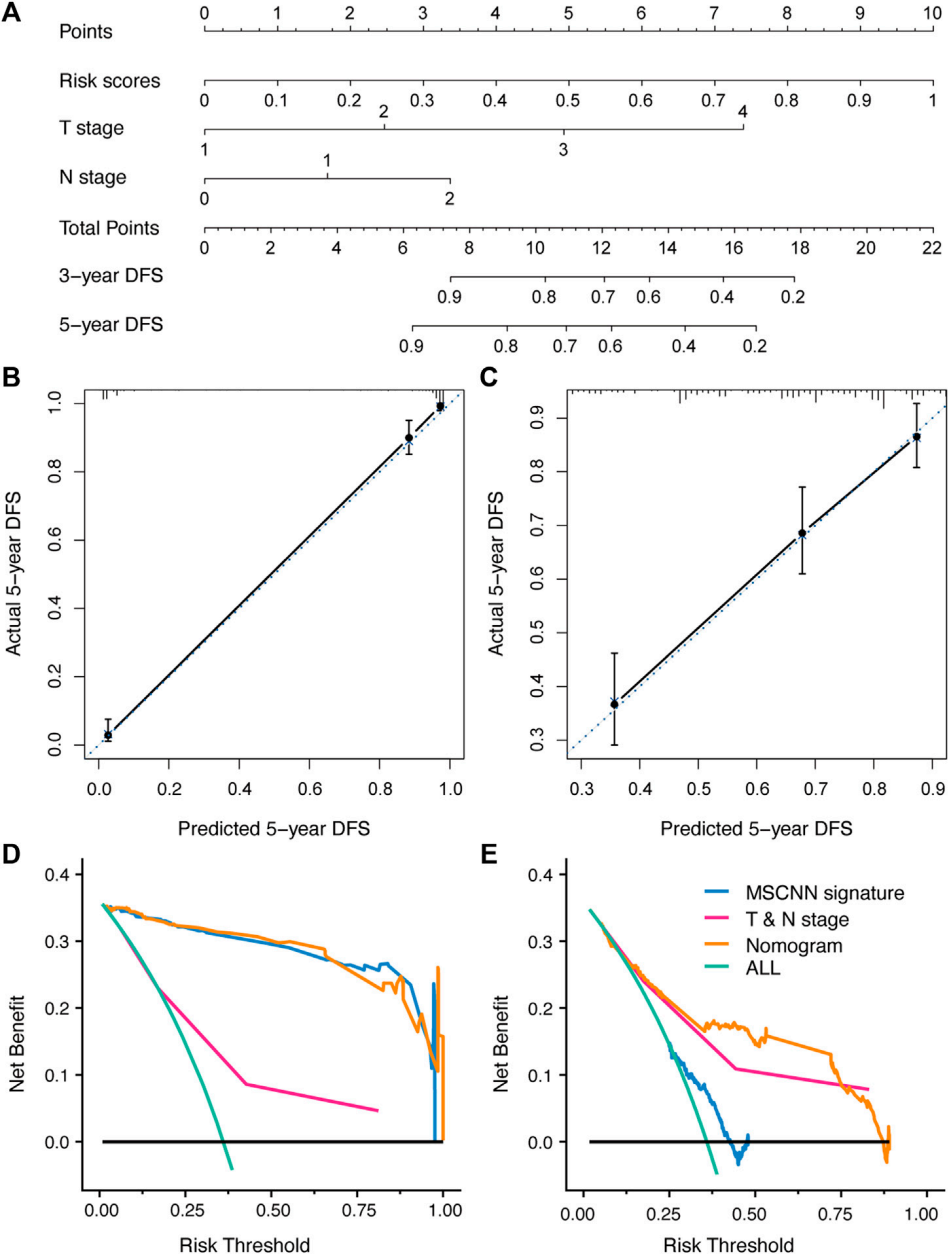
The developed nomogram incorporated CT signature with T & N stage **(A)**. Coordinates length for each prognostic factor was determined by the coefficients of the cox regression model. For each patient, the total score was calculated with all variable scores. The probability of DFS was derived from the mapping relationship between the evaluation results and total score on specified patient survival time. **(B,C)** Calibration curves of nomogram for 5 years DFS in the development and validation cohort. **(D,E)** Decision curve analysis for nomogram established in the development and validation cohort.

According to the multivariable analysis, the Cox regression model which incorporated CT signature, T stage and N stage was developed and displayed as a CT signature based nomogram (Figure 3A). The calibration curve of the radiomics nomogram showed good concordance between the prediction and the actual DFS survival (Figures 3B,C). DCA curve showed that nomogram achieved better net benefit compared with TNM-stage only (Figures 3E,F).

### Visualization for the Deep Features From Radiomics

**FIGURE 4:**
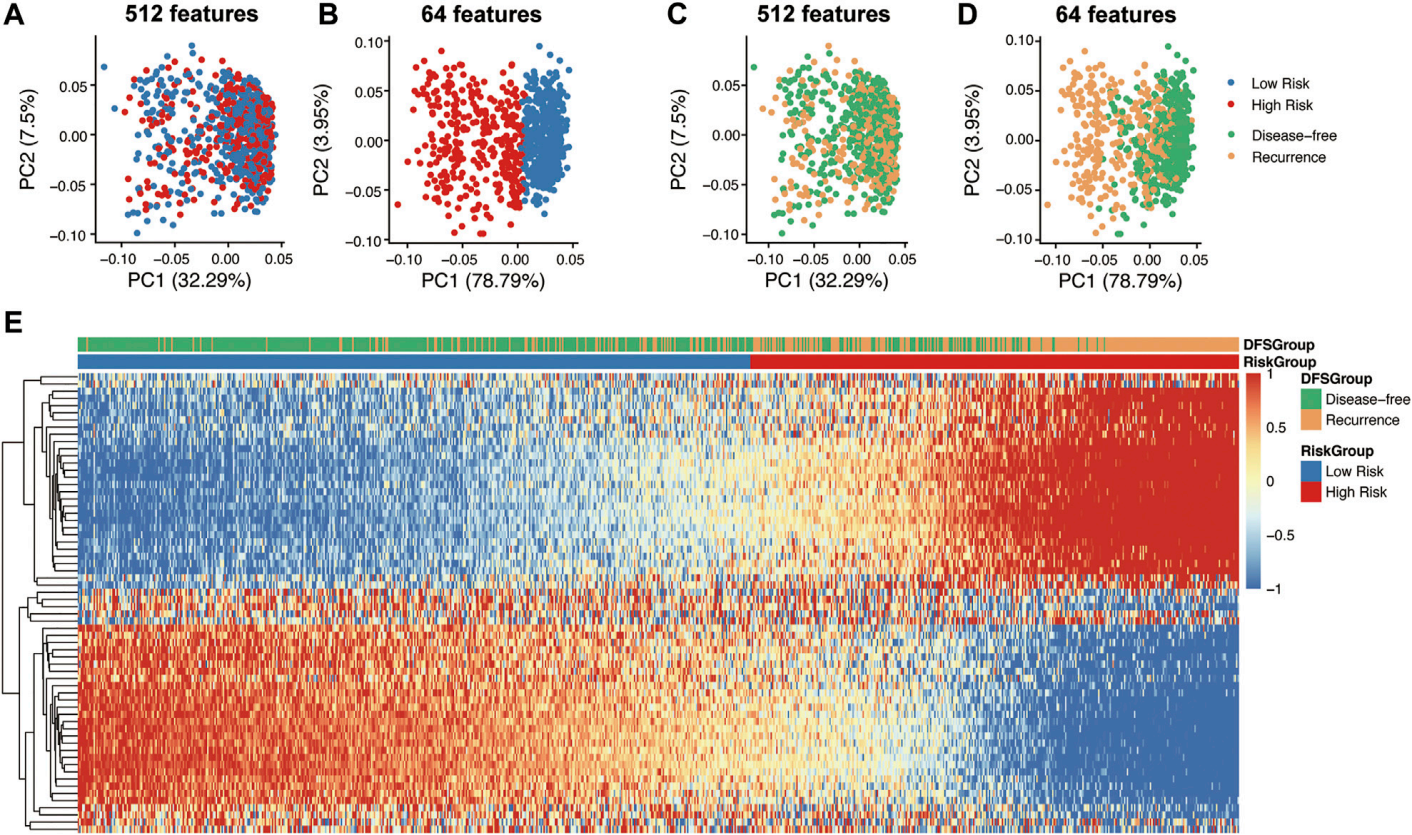
Dimension reduction for visualization and correlation analysis of deep CT features. Principle component analysis (PCA) on the 512 features of the ResNet34 network **(A,C)** and 64 features (CT feature) of hidden notes of the FC network **(B,D)**. Correlation heatmap between 64 deep CT features and prognostic difference group **(E)**.

Deep features were extracted from the output of the ResNet34 network and hidden notes of the FC layer. 512 features were exported from the ResNet34 network for each CT image, and then the 64 features most related to tumor recurrence and disease-free survival were extracted from the hidden layer. PCA analysis showed deep features from the RseNet34 network were not enough to accurately divide patients into high risk groups and low risk groups (Figures 4A,B). However, recurrence related 64 features extracted from the hidden layers achieved distinct classification (Figures 4C,D). Unsupervised clustering of 64 deep CT features displayed in the heatmap showed that these deep CT features were significantly highly correlated with high and low risk subgroups of CRC patients (Figure 4E).

### Pathway Analysis of Radiomics Risk Group and Deep Features

**FIGURE 5:**
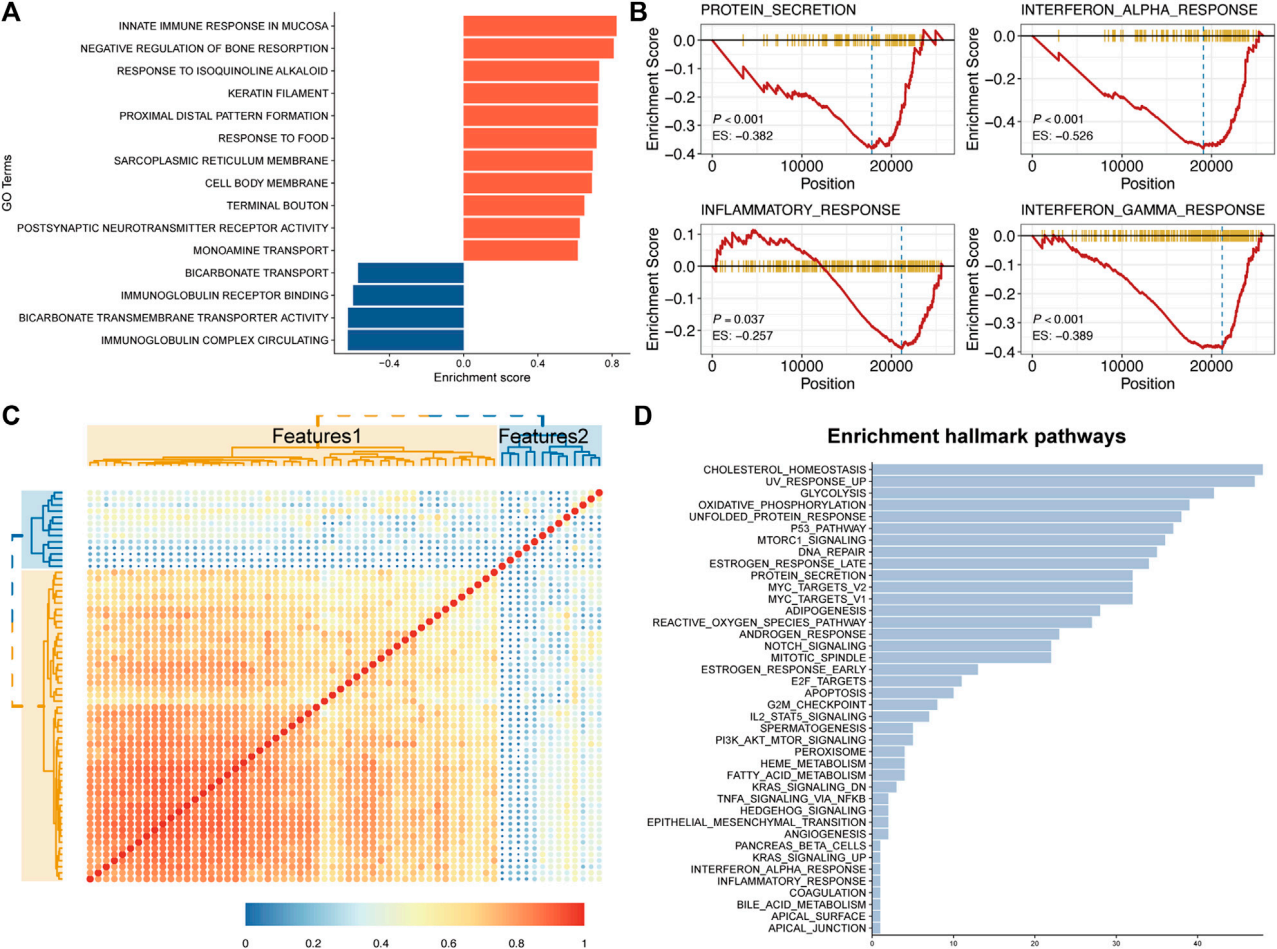
Global gene set pathway analysis. **(A)** Gene Ontology pathway enrichment analysis between CT signatures and RNA-Seq expression. **(B)** GSEA showed several Immune related pathways were downregulated in high CT signature patients. **(C,D)** Correlation between 64 deep CT features and their enrichment hallmark pathways.

To further explore the biological interpretability of deep CT features from the MSCNN model, Gene Ontology analysis of the different groups and the GSEA showed significant enrichment of immune pathways (Figure 5A) such as Interferon alpha response (*p* < 0.001), Interferon Gamma Response (*p* < 0.001) and Inflammatory response (*p* = 0.037) (Figure 5B). Significantly differential expression genes of risk groups were shown in Supplementary Figure S3. Besides, correlation analysis of the 64 deep CT features (Figure 5C) found that most of these features were highly correlated. Their further correlation analysis with the hallmark pathways was performed to explore the biological mechanism of the MSCNN model. Hallmark pathways were selected according to significant association with those deep CT features, and the result showed those features had a significant enrichment in some metabolism and proliferative pathways (Figure 5D).

## Discussion

In this study, we proposed a deep learning based end-to-end method to predict prognosis of colorectal cancer patients after tumor resection surgery from CT images. Our deep learning model successfully screened out high tumor recurrence risk patients with significant prognostic differences from the others. Univariable and multivariable analyses showed that CT signature was an independent factor for CRC patient survival prediction. By incorporating CT signature and clinical risk factors, we built a nomogram that can facilitate the risk prediction for colorectal cancer patients. Correlation analysis with genomic data indicated that high risk patients showed downregulation of immune pathways and deep CT features learned by MSCNN model were significantly enriched in some metabolism and proliferative pathways.

Traditional prognostic analysis based on genetic testing can obtain good performance as expression of several genes were highly related with patient’s tumor progression (Kandimalla et al., 2018; Sveen et al., 2020). However high cost and long test time cycle limited its large-scale applications. Compared with genetic testing, CT imaging, a much cheaper non-invasive preoperative routine test for CRC patients to locate the tumor before the resection surgery, can provide more preoperative interventions. Our study was based on deep learning model which focused detailed and deeper information of CT images and acquired good performance in prognostic prediction for CRC patients.

Deep learning model with CNN can learn the features of CT images from low to high dimensions and their correlation (Yamashita et al., 2018), which may be the key reason for high performance in image analysis. Since most previous CT image based prognostic research have only used pretrained deep learning to extract images features, subsequent analysis required subjective screening of these features to build the machine learning model again (Huang et al., 2020; Park et al., 2021; Liu et al., 2022). Besides, they did not consider the special characteristics of medical images which mean generic pretrained deep learning models were not suitable. Our MSCNN model was an end-to-end method to quickly predict the prognosis for CRC patients with CT images, which can also reduce the subjectivity of human selection of image characteristics. In addition, the percentage of tumors in CT images is often small, accounting for only about 1–5%, which makes it difficult for ordinary CNN models to learn the key information of CT images. Based on the idea of multi-instance learning in pathology research (Bilal et al., 2021; Sirinukunwattana et al., 2021), our MSCNN model considered both full-image and local detail information of CT images by cropping and deflating the ROI region, making the prognostic predicting of our model more comprehensive and accurate.

Recent rapid development of deep learning has generated a series of CNN based studies for radiographic analysis, like treatment response predicting (Xu et al., 2019; Lu et al., 2021) and detection of Synchronous Peritoneal Carcinomatosis (Yuan et al., 2020). However, few of them considered the interpretation of their deep learning models, making it hard for clinicians to be convinced of their findings. Our study not only visualized the process of classifying CRC patients in high and low risk groups, but also found that the CT signature of our MSCNN model was significantly correlated with several immune pathways. Meanwhile, our results found that deep CT features showed significant enrichment in some metabolic proliferative pathways which was consistent with previous studies (Kandimalla et al., 2019; Cai et al., 2020; La Vecchia and Sebastián, 2020).

However, despite satisfactory results with sufficient biological interpretation, our study still has some limitations. First, a prospective study was needed to further confirm and optimize our model. In our study, all patients included are from one single center, which may cause bias for the model validation. In addition, our CT images for prognosis predicting need manual ROI segmentation which is time-consuming and seriously affects the applicability of our model. This can be achieved by object detection through deep learning with enough data. In this way, the ROI can be directly learned from the model without manual sketching.

In conclusion, our study demonstrated that deep learning with CT images can be effectively applied to cancer recurrence prediction. By incorporating clinical factors, more accurate results can be achieved than just routine TNM staging. Correlation analysis with gene expression data showed that deep CT features captured by our model did have a biological meaning which gave credibility to our MSCNN model.

## Data Availability

The raw data supporting the conclusions of this study are available for reasonable request from the corresponding authors (FG). The CT images data are not public as they contain some private information of our patients. We only used gene expression data (but not microarray/deep sequencing) for pathway analysis to enhance the interpretability of the main results. Gene expression data of the ICGC-ARGO project are not public available currently and will be released on the website of the project (https://www.icgc-argo.org/page/114/cgcc) by ICGC after the milestone.
