# Supplementary material for: Multi-Size Deep Learning Based Preoperative Computed Tomography Signature for Prognosis Prediction of Colorectal Cancer
Source: Front Genet. 2022 May 12;13:880093. doi: 10.3389/fgene.2022.880093 (PMC9133721; doi:10.3389/fgene.2022.880093)
Supplement: Supplementary file 3 [file DataSheet1.docx]

Supplementary materials


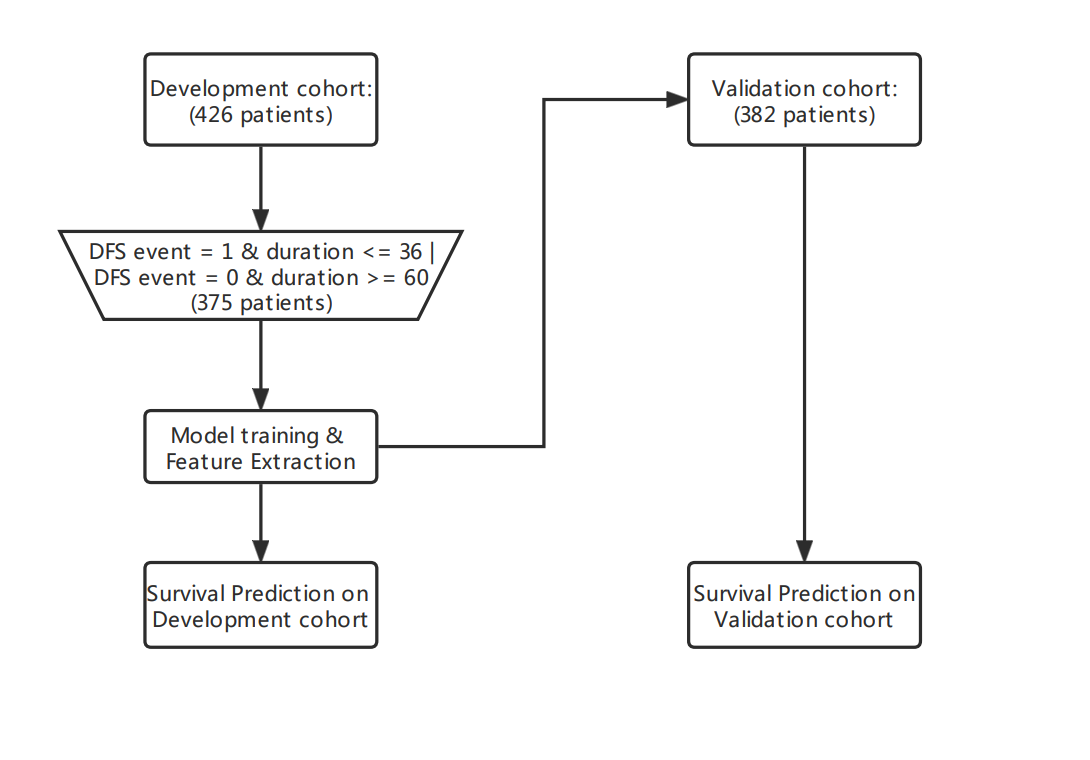


Supplementary Figure 1. Schematic flow chart of the study procedure


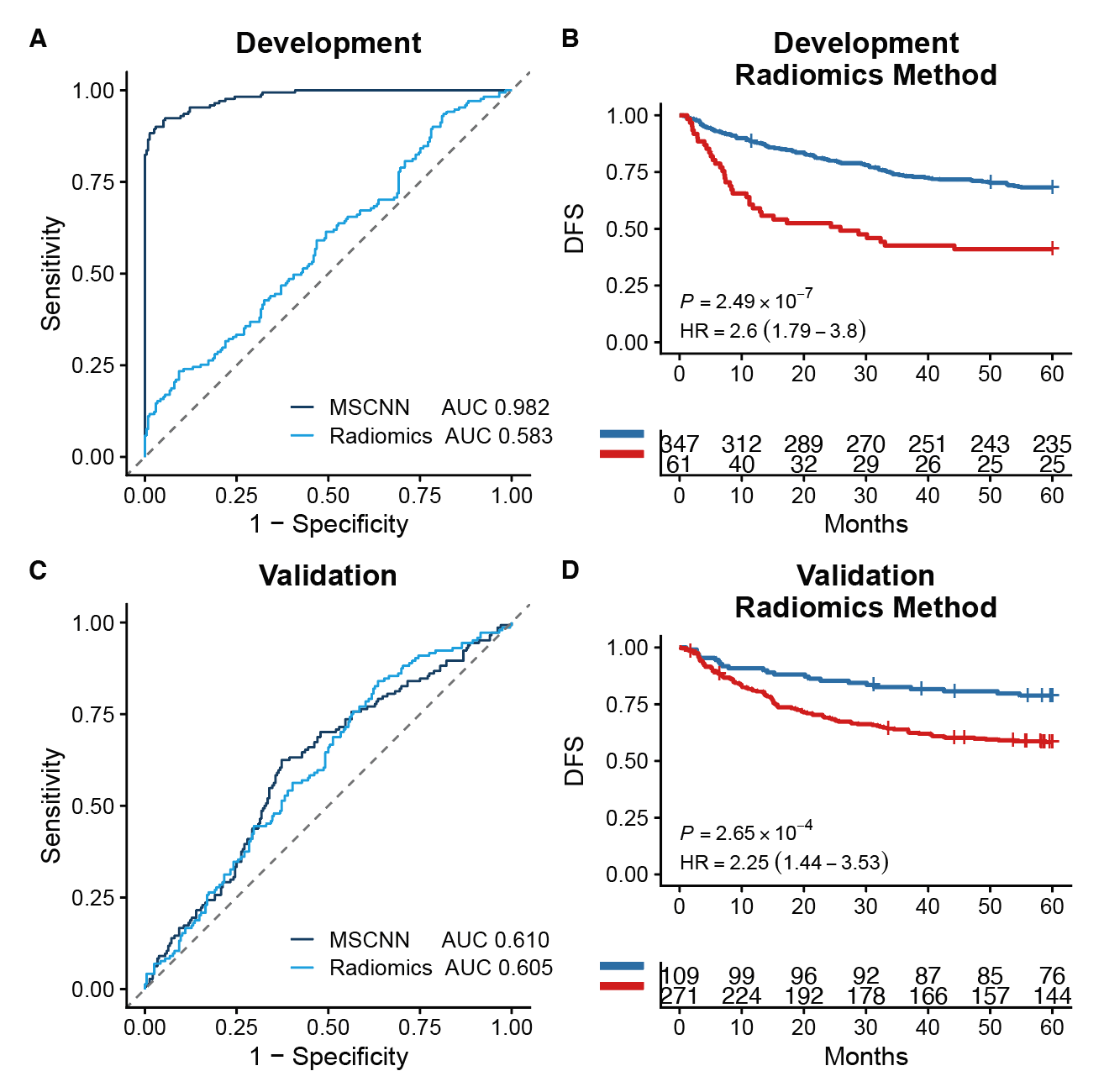


Supplementary Figure 2. MSDL Method vs. Radiomics Method. Kaplan-Meier curves for high recurrence risk and low recurrence risk patients predicted by Radiomics method in the development cohort (B) and validation cohort (D). Comparison between


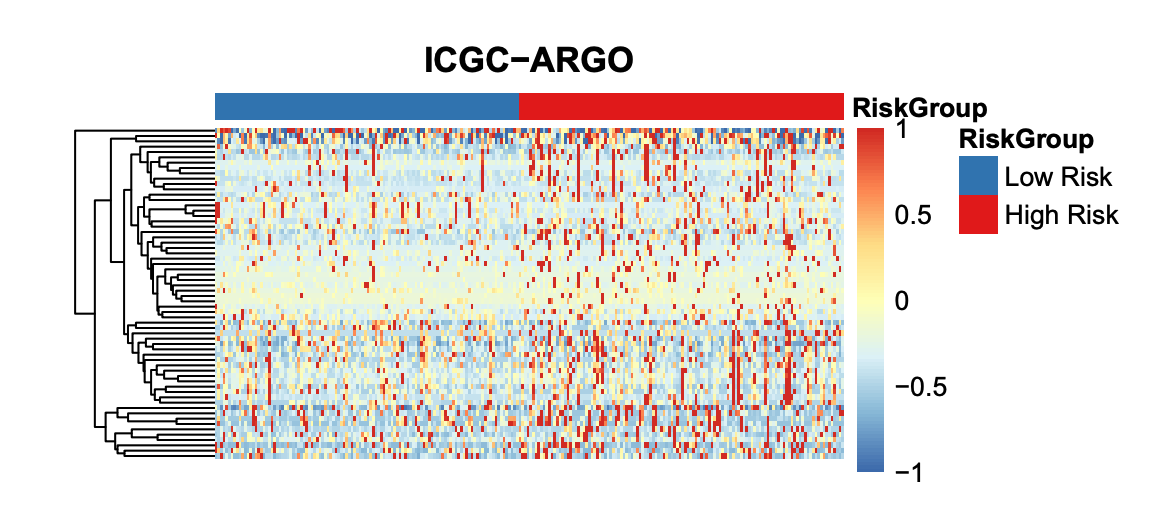


Supplementary Figure 3. Heatmap of different expression genes between high and low risk group

Supplementary Table 1. The risk groups and CT signature of CRC patients in this study.

| TuominID | Cohort | CT-signature | Risk_group | Gene-Data |
| --- | --- | --- | --- | --- |
| A028 | Development | 0.9994 | High Risk | No |
| A029 | Development | 0.9999 | High Risk | No |
| A055 | Development | 0.0431 | Low Risk | No |
| A057 | Development | 0.9998 | High Risk | No |
| A054 | Development | 0.0361 | Low Risk | No |
| A058 | Development | 0.0086 | Low Risk | No |
| A051 | Development | 0.0629 | Low Risk | No |
| A062 | Development | 0.5374 | High Risk | No |
| A063 | Development | 0.0434 | Low Risk | No |
| A064 | Development | 0.2336 | Low Risk | No |
| A065 | Development | 0.4640 | Low Risk | No |
| A056 | Development | 0.0642 | Low Risk | No |
| A066 | Development | 0.9998 | High Risk | No |
| A060 | Development | 1.0000 | High Risk | No |
| A061 | Development | 0.0327 | Low Risk | No |
| A030 | Development | 0.9997 | High Risk | No |
| A031 | Development | 0.0473 | Low Risk | No |
| A033 | Development | 0.0544 | Low Risk | No |
| A032 | Development | 0.1827 | Low Risk | No |
| A034 | Development | 0.2906 | Low Risk | No |
| A036 | Development | 0.9994 | High Risk | No |
| A039 | Development | 0.9998 | High Risk | No |
| A040 | Development | 0.0403 | Low Risk | No |
| A041 | Development | 0.9997 | High Risk | No |
| A043 | Development | 0.9991 | High Risk | No |
| A035 | Development | 0.9991 | High Risk | No |
| A037 | Development | 0.0800 | Low Risk | No |
| A046 | Development | 0.4452 | Low Risk | No |
| A048 | Development | 0.1267 | Low Risk | No |
| A042 | Development | 0.1322 | Low Risk | No |
| A044 | Development | 0.0280 | Low Risk | No |
| A045 | Development | 0.5189 | High Risk | No |
| A047 | Development | 0.9998 | High Risk | No |
| A050 | Development | 0.0788 | Low Risk | No |
| A053 | Development | 0.9998 | High Risk | No |
| A052 | Development | 0.9999 | High Risk | No |
| A067 | Development | 0.9983 | High Risk | No |
| A106 | Development | 0.0393 | Low Risk | No |
| A107 | Development | 0.4767 | Low Risk | No |
| A111 | Development | 0.0179 | Low Risk | Yes |
| A112 | Development | 0.2121 | Low Risk | No |
| A113 | Development | 0.9980 | High Risk | Yes |
| A114 | Development | 0.2132 | Low Risk | No |
| A110 | Development | 0.0265 | Low Risk | No |
| A109 | Development | 0.0289 | Low Risk | No |
| A117 | Development | 0.0244 | Low Risk | No |
| A119 | Development | 0.0335 | Low Risk | Yes |
| A116 | Development | 0.1758 | Low Risk | No |
| A120 | Development | 0.0277 | Low Risk | No |
| A122 | Development | 0.0193 | Low Risk | No |
| A001 | Development | 0.0596 | Low Risk | No |
| A002 | Development | 1.0000 | High Risk | Yes |
| A121 | Development | 0.9997 | High Risk | No |
| A118 | Development | 0.0196 | Low Risk | No |
| A071 | Development | 0.0355 | Low Risk | No |
| A070 | Development | 0.0297 | Low Risk | No |
| A069 | Development | 1.0000 | High Risk | No |
| A072 | Development | 0.0285 | Low Risk | No |
| A068 | Development | 0.9999 | High Risk | No |
| A074 | Development | 0.0396 | Low Risk | No |
| A077 | Development | 0.0380 | Low Risk | No |
| A076 | Development | 0.9965 | High Risk | No |
| A078 | Development | 0.0720 | Low Risk | No |
| A073 | Development | 0.0103 | Low Risk | No |
| A079 | Development | 0.0484 | Low Risk | No |
| A080 | Development | 0.9948 | High Risk | No |
| A075 | Development | 0.4236 | Low Risk | No |
| A083 | Development | 0.9966 | High Risk | No |
| A085 | Development | 0.0409 | Low Risk | No |
| A082 | Development | 0.0289 | Low Risk | No |
| A086 | Development | 0.0245 | Low Risk | No |
| A087 | Development | 0.0763 | Low Risk | No |
| A084 | Development | 0.0266 | Low Risk | No |
| A088 | Development | 0.0284 | Low Risk | No |
| A089 | Development | 0.0368 | Low Risk | No |
| A081 | Development | 0.9438 | High Risk | No |
| A092 | Development | 0.0364 | Low Risk | No |
| A094 | Development | 0.9999 | High Risk | No |
| A091 | Development | 0.9996 | High Risk | No |
| A096 | Development | 0.9999 | High Risk | No |
| A097 | Development | 0.0433 | Low Risk | No |
| A095 | Development | 0.9997 | High Risk | No |
| A099 | Development | 0.0949 | Low Risk | No |
| A100 | Development | 0.1271 | Low Risk | No |
| A098 | Development | 0.1813 | Low Risk | No |
| A102 | Development | 0.0300 | Low Risk | No |
| A101 | Development | 0.0384 | Low Risk | No |
| A103 | Development | 0.8581 | High Risk | No |
| A104 | Development | 0.0366 | Low Risk | No |
| A124 | Development | 0.9999 | High Risk | No |
| A125 | Development | 0.0224 | Low Risk | No |
| A123 | Development | 0.0203 | Low Risk | Yes |
| A126 | Development | 0.2738 | Low Risk | No |
| A022 | Development | 0.0562 | Low Risk | Yes |
| A142 | Development | 0.0198 | Low Risk | No |
| A145 | Development | 0.1086 | Low Risk | Yes |
| A143 | Development | 0.9995 | High Risk | No |
| A144 | Development | 0.9987 | High Risk | Yes |
| A146 | Development | 1.0000 | High Risk | Yes |
| A141 | Development | 0.0202 | Low Risk | No |
| A147 | Development | 0.0199 | Low Risk | Yes |
| A150 | Development | 0.0192 | Low Risk | No |
| A153 | Development | 0.0758 | Low Risk | Yes |
| A154 | Development | 0.0720 | Low Risk | Yes |
| A155 | Development | 0.0378 | Low Risk | Yes |
| A148 | Development | 0.0702 | Low Risk | Yes |
| A151 | Development | 0.0512 | Low Risk | Yes |
| A149 | Development | 0.2958 | Low Risk | Yes |
| A152 | Development | 0.0945 | Low Risk | Yes |
| A127 | Development | 0.1107 | Low Risk | No |
| A129 | Development | 0.2952 | Low Risk | No |
| A130 | Development | 0.0447 | Low Risk | No |
| A128 | Development | 0.1325 | Low Risk | No |
| A131 | Development | 0.0331 | Low Risk | No |
| A049 | Development | 1.0000 | High Risk | No |
| A132 | Development | 0.1006 | Low Risk | No |
| A003 | Development | 0.0155 | Low Risk | No |
| A133 | Development | 0.9996 | High Risk | No |
| A134 | Development | 0.9993 | High Risk | No |
| A135 | Development | 0.0324 | Low Risk | No |
| A137 | Development | 0.1152 | Low Risk | Yes |
| A136 | Development | 0.9990 | High Risk | Yes |
| A139 | Development | 0.0394 | Low Risk | No |
| A138 | Development | 0.0388 | Low Risk | No |
| A140 | Development | 0.9994 | High Risk | No |
| A157 | Development | 0.4539 | Low Risk | No |
| A158 | Development | 0.9987 | High Risk | No |
| A159 | Development | 0.9999 | High Risk | Yes |
| A160 | Development | 0.9964 | High Risk | Yes |
| A162 | Development | 0.0678 | Low Risk | Yes |
| A156 | Development | 0.0529 | Low Risk | Yes |
| A230 | Development | 0.0571 | Low Risk | No |
| A231 | Development | 0.1473 | Low Risk | No |
| A232 | Development | 0.9999 | High Risk | No |
| A233 | Development | 0.0119 | Low Risk | Yes |
| A234 | Development | 0.9996 | High Risk | Yes |
| A236 | Development | 0.0282 | Low Risk | Yes |
| A239 | Development | 0.0704 | Low Risk | Yes |
| A240 | Development | 0.0364 | Low Risk | Yes |
| A241 | Development | 0.0456 | Low Risk | No |
| A237 | Development | 1.0000 | High Risk | No |
| A238 | Development | 0.9999 | High Risk | Yes |
| A243 | Development | 0.0357 | Low Risk | No |
| A244 | Development | 0.0157 | Low Risk | No |
| A242 | Development | 0.0372 | Low Risk | No |
| A013 | Development | 0.2000 | Low Risk | No |
| A014 | Development | 0.0655 | Low Risk | Yes |
| A235 | Development | 0.8138 | High Risk | Yes |
| A012 | Development | 0.9989 | High Risk | Yes |
| A246 | Development | 1.0000 | High Risk | No |
| A253 | Development | 0.0667 | Low Risk | No |
| A254 | Development | 0.0321 | Low Risk | No |
| A245 | Development | 0.0415 | Low Risk | No |
| A258 | Development | 1.0000 | High Risk | No |
| A247 | Development | 0.0610 | Low Risk | No |
| A256 | Development | 1.0000 | High Risk | No |
| A257 | Development | 0.9999 | High Risk | No |
| A259 | Development | 0.0775 | Low Risk | No |
| A260 | Development | 0.0580 | Low Risk | No |
| A023 | Development | 0.0572 | Low Risk | No |
| A251 | Development | 0.0266 | Low Risk | Yes |
| A250 | Development | 0.9991 | High Risk | No |
| A252 | Development | 0.9996 | High Risk | Yes |
| A255 | Development | 0.9999 | High Risk | Yes |
| A248 | Development | 0.0467 | Low Risk | Yes |
| A163 | Development | 0.9991 | High Risk | Yes |
| A164 | Development | 0.0667 | Low Risk | Yes |
| A165 | Development | 0.9997 | High Risk | Yes |
| A166 | Development | 0.9999 | High Risk | Yes |
| A168 | Development | 0.9999 | High Risk | Yes |
| A006 | Development | 0.0651 | Low Risk | Yes |
| A170 | Development | 0.9733 | High Risk | No |
| A171 | Development | 0.0482 | Low Risk | No |
| A172 | Development | 0.0228 | Low Risk | No |
| A174 | Development | 0.9998 | High Risk | No |
| A173 | Development | 0.0347 | Low Risk | Yes |
| A176 | Development | 0.0338 | Low Risk | No |
| A175 | Development | 0.0452 | Low Risk | No |
| A177 | Development | 0.1485 | Low Risk | No |
| A167 | Development | 0.0799 | Low Risk | Yes |
| A161 | Development | 0.0296 | Low Risk | Yes |
| A169 | Development | 0.0349 | Low Risk | No |
| A180 | Development | 0.1290 | Low Risk | Yes |
| A181 | Development | 0.0276 | Low Risk | No |
| A183 | Development | 0.0429 | Low Risk | No |
| A184 | Development | 0.0329 | Low Risk | No |
| A182 | Development | 0.0264 | Low Risk | No |
| A186 | Development | 0.1366 | Low Risk | No |
| A178 | Development | 0.1284 | Low Risk | No |
| A008 | Development | 0.0196 | Low Risk | Yes |
| A189 | Development | 0.0666 | Low Risk | No |
| A190 | Development | 0.9998 | High Risk | No |
| A191 | Development | 0.9999 | High Risk | No |
| A192 | Development | 0.0339 | Low Risk | No |
| A193 | Development | 0.1784 | Low Risk | Yes |
| A194 | Development | 0.9997 | High Risk | No |
| A185 | Development | 0.0354 | Low Risk | No |
| A187 | Development | 0.9998 | High Risk | No |
| A188 | Development | 0.9996 | High Risk | No |
| A196 | Development | 0.9983 | High Risk | No |
| A195 | Development | 0.0504 | Low Risk | No |
| A199 | Development | 0.0133 | Low Risk | Yes |
| A202 | Development | 0.0216 | Low Risk | No |
| A201 | Development | 0.0326 | Low Risk | No |
| A197 | Development | 0.0142 | Low Risk | No |
| A204 | Development | 0.9993 | High Risk | Yes |
| A205 | Development | 0.0367 | Low Risk | No |
| A206 | Development | 0.0607 | Low Risk | No |
| A021 | Development | 0.9976 | High Risk | No |
| A208 | Development | 1.0000 | High Risk | No |
| A209 | Development | 0.9998 | High Risk | No |
| A210 | Development | 1.0000 | High Risk | No |
| A212 | Development | 0.0602 | Low Risk | No |
| A211 | Development | 0.0432 | Low Risk | No |
| A214 | Development | 0.0361 | Low Risk | No |
| A216 | Development | 0.2100 | Low Risk | Yes |
| A010 | Development | 0.0453 | Low Risk | Yes |
| A207 | Development | 0.2839 | Low Risk | No |
| A213 | Development | 0.0508 | Low Risk | No |
| A219 | Development | 0.0331 | Low Risk | No |
| A215 | Development | 0.0210 | Low Risk | Yes |
| A217 | Development | 1.0000 | High Risk | No |
| A220 | Development | 0.9995 | High Risk | No |
| A011 | Development | 0.0887 | Low Risk | Yes |
| A221 | Development | 0.9994 | High Risk | Yes |
| A222 | Development | 0.0220 | Low Risk | No |
| A218 | Development | 0.0856 | Low Risk | Yes |
| A223 | Development | 0.9986 | High Risk | Yes |
| A224 | Development | 1.0000 | High Risk | Yes |
| A226 | Development | 0.0347 | Low Risk | Yes |
| A225 | Development | 0.0343 | Low Risk | Yes |
| A227 | Development | 0.0339 | Low Risk | No |
| A228 | Development | 0.9997 | High Risk | No |
| A229 | Development | 0.4415 | Low Risk | No |
| A261 | Development | 0.0445 | Low Risk | No |
| A262 | Development | 0.0313 | Low Risk | Yes |
| A263 | Development | 0.1385 | Low Risk | No |
| A331 | Development | 0.0391 | Low Risk | No |
| A332 | Development | 0.9982 | High Risk | No |
| A324 | Development | 0.9999 | High Risk | No |
| A333 | Development | 0.0297 | Low Risk | No |
| A334 | Development | 0.0221 | Low Risk | No |
| A335 | Development | 0.0783 | Low Risk | No |
| C073 | Development | 0.0208 | Low Risk | No |
| B086 | Development | 0.0242 | Low Risk | No |
| C074 | Development | 0.6907 | High Risk | No |
| B087 | Development | 0.0886 | Low Risk | No |
| A329 | Development | 0.0671 | Low Risk | No |
| C072 | Development | 0.0109 | Low Risk | No |
| A265 | Development | 0.0594 | Low Risk | Yes |
| A266 | Development | 0.0243 | Low Risk | Yes |
| A267 | Development | 0.1873 | Low Risk | No |
| A015 | Development | 0.0180 | Low Risk | Yes |
| A016 | Development | 0.0366 | Low Risk | Yes |
| A269 | Development | 0.9996 | High Risk | No |
| A276 | Development | 0.9995 | High Risk | Yes |
| A277 | Development | 0.0161 | Low Risk | Yes |
| A278 | Development | 1.0000 | High Risk | Yes |
| A275 | Development | 0.9997 | High Risk | No |
| A264 | Development | 0.9930 | High Risk | No |
| A279 | Development | 0.0167 | Low Risk | No |
| A017 | Development | 0.0681 | Low Risk | Yes |
| A280 | Development | 0.1405 | Low Risk | Yes |
| A281 | Development | 0.0217 | Low Risk | Yes |
| A282 | Development | 0.0107 | Low Risk | Yes |
| A283 | Development | 0.0145 | Low Risk | Yes |
| A270 | Development | 0.9999 | High Risk | No |
| A271 | Development | 0.0322 | Low Risk | No |
| A272 | Development | 0.9999 | High Risk | No |
| A273 | Development | 0.8414 | High Risk | No |
| A274 | Development | 0.0178 | Low Risk | No |
| A285 | Development | 1.0000 | High Risk | Yes |
| A286 | Development | 0.9825 | High Risk | Yes |
| A287 | Development | 0.0436 | Low Risk | Yes |
| A284 | Development | 0.0353 | Low Risk | Yes |
| A294 | Development | 0.0407 | Low Risk | Yes |
| A295 | Development | 0.0199 | Low Risk | Yes |
| A293 | Development | 0.0417 | Low Risk | Yes |
| A297 | Development | 0.0424 | Low Risk | Yes |
| A296 | Development | 0.9960 | High Risk | Yes |
| A289 | Development | 0.4113 | Low Risk | No |
| A290 | Development | 0.0208 | Low Risk | Yes |
| A298 | Development | 0.9995 | High Risk | Yes |
| A300 | Development | 0.1075 | Low Risk | Yes |
| A301 | Development | 0.0287 | Low Risk | Yes |
| A291 | Development | 0.6541 | High Risk | Yes |
| A299 | Development | 0.1238 | Low Risk | Yes |
| A292 | Development | 0.1395 | Low Risk | No |
| A304 | Development | 0.0084 | Low Risk | Yes |
| A303 | Development | 0.9999 | High Risk | Yes |
| A307 | Development | 0.0609 | Low Risk | No |
| A308 | Development | 0.0304 | Low Risk | Yes |
| A302 | Development | 0.0200 | Low Risk | Yes |
| A314 | Development | 0.0809 | Low Risk | No |
| A288 | Development | 0.9997 | High Risk | No |
| A313 | Development | 0.0294 | Low Risk | No |
| A315 | Development | 0.0544 | Low Risk | No |
| A312 | Development | 0.9998 | High Risk | Yes |
| A018 | Development | 0.7554 | High Risk | No |
| A318 | Development | 0.1923 | Low Risk | Yes |
| A316 | Development | 0.9999 | High Risk | Yes |
| A320 | Development | 0.0336 | Low Risk | Yes |
| A317 | Development | 0.9982 | High Risk | No |
| A319 | Development | 0.9945 | High Risk | Yes |
| A020 | Development | 0.9997 | High Risk | Yes |
| A309 | Development | 0.7788 | High Risk | Yes |
| A311 | Development | 0.7510 | High Risk | Yes |
| A322 | Development | 0.1681 | Low Risk | Yes |
| A019 | Development | 1.0000 | High Risk | Yes |
| A310 | Development | 0.9998 | High Risk | Yes |
| A328 | Development | 0.9995 | High Risk | Yes |
| A330 | Development | 0.9999 | High Risk | No |
| A326 | Development | 0.9999 | High Risk | Yes |
| A323 | Development | 1.0000 | High Risk | Yes |
| A325 | Development | 1.0000 | High Risk | Yes |
| A321 | Development | 0.4079 | Low Risk | Yes |
| A327 | Development | 0.0862 | Low Risk | Yes |
| C075 | Development | 0.9996 | High Risk | No |
| B090 | Development | 0.8387 | High Risk | No |
| C076 | Development | 0.9999 | High Risk | No |
| B091 | Development | 0.9999 | High Risk | No |
| B088 | Development | 0.0468 | Low Risk | No |
| C158 | Development | 0.0616 | Low Risk | No |
| C160 | Development | 0.0605 | Low Risk | No |
| B108 | Development | 0.1093 | Low Risk | No |
| C162 | Development | 0.1007 | Low Risk | No |
| C163 | Development | 0.0530 | Low Risk | No |
| C169 | Development | 0.9999 | High Risk | No |
| C167 | Development | 0.1615 | Low Risk | No |
| B110 | Development | 0.0207 | Low Risk | No |
| B111 | Development | 0.9998 | High Risk | No |
| B112 | Development | 0.0261 | Low Risk | No |
| C121 | Development | 0.9994 | High Risk | No |
| A339 | Development | 0.9999 | High Risk | No |
| C171 | Development | 0.0251 | Low Risk | No |
| C170 | Development | 0.0514 | Low Risk | No |
| C161 | Development | 0.0219 | Low Risk | No |
| A340 | Development | 0.9997 | High Risk | No |
| C173 | Development | 0.9999 | High Risk | No |
| C174 | Development | 0.0529 | Low Risk | No |
| B106 | Development | 0.0363 | Low Risk | No |
| C155 | Development | 0.0446 | Low Risk | No |
| C157 | Development | 0.0794 | Low Risk | No |
| A338 | Development | 0.9999 | High Risk | Yes |
| A349 | Development | 0.9998 | High Risk | No |
| A341 | Development | 0.1177 | Low Risk | No |
| A342 | Development | 0.9998 | High Risk | Yes |
| C178 | Development | 0.8561 | High Risk | No |
| B114 | Development | 0.9999 | High Risk | No |
| A350 | Development | 1.0000 | High Risk | No |
| A352 | Development | 0.9998 | High Risk | Yes |
| A347 | Development | 0.0578 | Low Risk | No |
| A351 | Development | 1.0000 | High Risk | Yes |
| A354 | Development | 0.0400 | Low Risk | Yes |
| A355 | Development | 0.0518 | Low Risk | No |
| A356 | Development | 0.0183 | Low Risk | No |
| B117 | Development | 0.9999 | High Risk | No |
| C148 | Development | 0.9986 | High Risk | No |
| A348 | Development | 0.0710 | Low Risk | No |
| C077 | Development | 0.0552 | Low Risk | No |
| C078 | Development | 0.0201 | Low Risk | No |
| B089 | Development | 0.9954 | High Risk | No |
| C082 | Development | 0.0238 | Low Risk | No |
| C085 | Development | 0.9924 | High Risk | No |
| B085 | Development | 0.0315 | Low Risk | No |
| C086 | Development | 0.0428 | Low Risk | No |
| C087 | Development | 0.9995 | High Risk | No |
| C088 | Development | 0.0488 | Low Risk | No |
| C080 | Development | 0.0526 | Low Risk | No |
| B094 | Development | 0.9999 | High Risk | No |
| B093 | Development | 0.7890 | High Risk | No |
| C092 | Development | 0.0874 | Low Risk | No |
| C093 | Development | 0.0563 | Low Risk | No |
| C090 | Development | 0.0273 | Low Risk | No |
| C094 | Development | 0.0376 | Low Risk | No |
| B095 | Development | 0.0443 | Low Risk | No |
| C096 | Development | 0.0438 | Low Risk | No |
| B092 | Development | 0.1590 | Low Risk | No |
| C098 | Development | 0.0372 | Low Risk | No |
| B096 | Development | 0.9993 | High Risk | No |
| C099 | Development | 0.0388 | Low Risk | No |
| C101 | Development | 0.0408 | Low Risk | No |
| C100 | Development | 0.0345 | Low Risk | No |
| C091 | Development | 0.0292 | Low Risk | No |
| C104 | Development | 0.9996 | High Risk | No |
| B098 | Development | 0.0449 | Low Risk | No |
| C103 | Development | 0.0183 | Low Risk | No |
| C107 | Development | 0.9999 | High Risk | No |
| C108 | Development | 0.9998 | High Risk | No |
| C106 | Development | 0.0424 | Low Risk | No |
| C105 | Development | 0.9998 | High Risk | No |
| B100 | Development | 0.0594 | Low Risk | No |
| C112 | Development | 0.0513 | Low Risk | No |
| C111 | Development | 0.0439 | Low Risk | No |
| C109 | Development | 0.9998 | High Risk | No |
| C110 | Development | 0.0799 | Low Risk | No |
| B099 | Development | 0.0262 | Low Risk | No |
| C113 | Development | 0.8127 | High Risk | No |
| C115 | Development | 0.9997 | High Risk | No |
| C117 | Development | 0.9998 | High Risk | No |
| C081 | Development | 0.9999 | High Risk | No |
| C116 | Development | 0.0313 | Low Risk | No |
| C114 | Development | 0.9999 | High Risk | No |
| C120 | Development | 0.0302 | Low Risk | No |
| C123 | Development | 0.9999 | High Risk | No |
| C127 | Development | 0.1023 | Low Risk | No |
| A336 | Development | 0.9999 | High Risk | No |
| C128 | Development | 0.0363 | Low Risk | No |
| C136 | Development | 0.0320 | Low Risk | No |
| A337 | Development | 0.0199 | Low Risk | No |
| A268 | Development | 0.9999 | High Risk | No |
| B102 | Development | 0.0265 | Low Risk | No |
| B101 | Development | 0.0568 | Low Risk | No |
| B103 | Development | 0.9998 | High Risk | No |
| C138 | Development | 0.0181 | Low Risk | No |
| B104 | Development | 0.0617 | Low Risk | No |
| B105 | Development | 0.9993 | High Risk | No |
| C154 | Development | 0.0329 | Low Risk | No |
| C152 | Development | 0.0237 | Low Risk | No |
| C151 | Development | 0.9999 | High Risk | No |
| C156 | Development | 0.9999 | High Risk | No |
| B107 | Development | 0.5289 | High Risk | No |
| C153 | Development | 1.0000 | High Risk | No |
| C140 | Development | 0.9079 | High Risk | No |
| C141 | Development | 0.0623 | Low Risk | No |
| C143 | Development | 0.0540 | Low Risk | No |
| B120 | Validation | 0.9928 | High Risk | No |
| A361 | Validation | 0.9994 | High Risk | No |
| B122 | Validation | 0.0350 | Low Risk | No |
| B124 | Validation | 0.0447 | Low Risk | Yes |
| B123 | Validation | 0.3732 | Low Risk | No |
| A362 | Validation | 0.0126 | Low Risk | No |
| A364 | Validation | 0.5713 | High Risk | No |
| A357 | Validation | 0.1964 | Low Risk | Yes |
| A358 | Validation | 0.4958 | Low Risk | No |
| A360 | Validation | 0.8989 | High Risk | No |
| B118 | Validation | 0.5881 | High Risk | No |
| A359 | Validation | 0.0523 | Low Risk | Yes |
| B113 | Validation | 0.9974 | High Risk | No |
| B119 | Validation | 0.0676 | Low Risk | No |
| A249 | Validation | 0.9962 | High Risk | No |
| C046 | Validation | 0.0803 | Low Risk | No |
| C047 | Validation | 0.9209 | High Risk | No |
| C050 | Validation | 0.0711 | Low Risk | No |
| C048 | Validation | 0.9817 | High Risk | No |
| C049 | Validation | 0.8723 | High Risk | No |
| C043 | Validation | 0.1826 | Low Risk | No |
| C045 | Validation | 0.8562 | High Risk | No |
| C053 | Validation | 0.0318 | Low Risk | No |
| A389 | Validation | 0.2938 | Low Risk | No |
| C054 | Validation | 0.0248 | Low Risk | No |
| C059 | Validation | 0.6686 | High Risk | No |
| C055 | Validation | 0.0679 | Low Risk | No |
| C056 | Validation | 0.7912 | High Risk | No |
| C060 | Validation | 0.0861 | Low Risk | No |
| C051 | Validation | 0.7209 | High Risk | No |
| C052 | Validation | 0.0478 | Low Risk | No |
| C061 | Validation | 0.7232 | High Risk | No |
| C062 | Validation | 0.9953 | High Risk | No |
| C064 | Validation | 0.0583 | Low Risk | No |
| C057 | Validation | 0.0661 | Low Risk | No |
| C347 | Validation | 0.0360 | Low Risk | No |
| C065 | Validation | 0.0355 | Low Risk | No |
| C345 | Validation | 0.1431 | Low Risk | No |
| C351 | Validation | 0.3212 | Low Risk | No |
| C063 | Validation | 0.6984 | High Risk | No |
| A365 | Validation | 0.2726 | Low Risk | No |
| A368 | Validation | 0.0215 | Low Risk | No |
| B001 | Validation | 0.1845 | Low Risk | No |
| B002 | Validation | 0.9856 | High Risk | No |
| A343 | Validation | 0.9982 | High Risk | Yes |
| A367 | Validation | 0.4357 | Low Risk | No |
| A372 | Validation | 0.7820 | High Risk | Yes |
| A373 | Validation | 0.1519 | Low Risk | Yes |
| A371 | Validation | 0.9971 | High Risk | No |
| A370 | Validation | 0.9086 | High Risk | Yes |
| A374 | Validation | 0.1202 | Low Risk | No |
| C159 | Validation | 0.9947 | High Risk | No |
| B121 | Validation | 0.0233 | Low Risk | No |
| A369 | Validation | 0.1115 | Low Risk | No |
| A363 | Validation | 0.9922 | High Risk | No |
| B003 | Validation | 0.9859 | High Risk | No |
| A375 | Validation | 0.1021 | Low Risk | Yes |
| A376 | Validation | 0.9985 | High Risk | No |
| B004 | Validation | 0.1309 | Low Risk | No |
| A366 | Validation | 0.0978 | Low Risk | No |
| A377 | Validation | 0.3357 | Low Risk | Yes |
| A385 | Validation | 0.9338 | High Risk | Yes |
| A384 | Validation | 0.5829 | High Risk | No |
| A381 | Validation | 0.9457 | High Risk | No |
| B007 | Validation | 0.9983 | High Risk | No |
| C119 | Validation | 0.9019 | High Risk | No |
| A383 | Validation | 0.8568 | High Risk | Yes |
| B006 | Validation | 0.1269 | Low Risk | Yes |
| B012 | Validation | 0.0265 | Low Risk | No |
| B008 | Validation | 0.9813 | High Risk | No |
| B115 | Validation | 0.7476 | High Risk | No |
| B005 | Validation | 0.8857 | High Risk | Yes |
| B011 | Validation | 0.8731 | High Risk | Yes |
| B013 | Validation | 0.7493 | High Risk | No |
| B009 | Validation | 0.9667 | High Risk | No |
| B016 | Validation | 0.3905 | Low Risk | No |
| B014 | Validation | 0.3199 | Low Risk | Yes |
| B020 | Validation | 0.0893 | Low Risk | Yes |
| B015 | Validation | 0.6157 | High Risk | No |
| B017 | Validation | 0.1575 | Low Risk | Yes |
| B018 | Validation | 0.9572 | High Risk | Yes |
| B022 | Validation | 0.9941 | High Risk | Yes |
| B023 | Validation | 0.1804 | Low Risk | Yes |
| B021 | Validation | 0.8929 | High Risk | Yes |
| B024 | Validation | 0.9974 | High Risk | Yes |
| A379 | Validation | 0.1637 | Low Risk | No |
| A380 | Validation | 0.2590 | Low Risk | Yes |
| A378 | Validation | 0.8391 | High Risk | No |
| A382 | Validation | 0.0877 | Low Risk | No |
| A305 | Validation | 0.0725 | Low Risk | Yes |
| B029 | Validation | 0.4129 | Low Risk | Yes |
| B032 | Validation | 0.7404 | High Risk | Yes |
| B035 | Validation | 0.9716 | High Risk | Yes |
| B037 | Validation | 0.8772 | High Risk | Yes |
| B038 | Validation | 0.2891 | Low Risk | No |
| B040 | Validation | 0.0573 | Low Risk | Yes |
| B046 | Validation | 0.8984 | High Risk | Yes |
| B047 | Validation | 0.0390 | Low Risk | Yes |
| B025 | Validation | 0.1145 | Low Risk | No |
| B027 | Validation | 0.9340 | High Risk | No |
| B036 | Validation | 0.2595 | Low Risk | Yes |
| B049 | Validation | 0.9327 | High Risk | No |
| B041 | Validation | 0.9718 | High Risk | No |
| B048 | Validation | 0.2528 | Low Risk | Yes |
| B010 | Validation | 0.0349 | Low Risk | Yes |
| B028 | Validation | 0.0157 | Low Risk | No |
| B052 | Validation | 0.9994 | High Risk | No |
| B053 | Validation | 0.1791 | Low Risk | No |
| B055 | Validation | 0.0854 | Low Risk | No |
| B039 | Validation | 0.3811 | Low Risk | Yes |
| B051 | Validation | 0.7595 | High Risk | No |
| B054 | Validation | 0.9995 | High Risk | Yes |
| C236 | Validation | 0.0578 | Low Risk | No |
| B050 | Validation | 0.0956 | Low Risk | Yes |
| B056 | Validation | 0.9945 | High Risk | No |
| B057 | Validation | 0.9166 | High Risk | Yes |
| B058 | Validation | 0.0687 | Low Risk | No |
| B026 | Validation | 0.7345 | High Risk | Yes |
| B030 | Validation | 0.7081 | High Risk | No |
| B033 | Validation | 0.8428 | High Risk | Yes |
| B019 | Validation | 0.3754 | Low Risk | Yes |
| B031 | Validation | 0.4834 | Low Risk | No |
| B034 | Validation | 0.3869 | Low Risk | Yes |
| A353 | Validation | 0.7187 | High Risk | No |
| B061 | Validation | 0.9941 | High Risk | No |
| B062 | Validation | 0.0581 | Low Risk | No |
| B067 | Validation | 0.9153 | High Risk | Yes |
| B071 | Validation | 0.9453 | High Risk | Yes |
| A346 | Validation | 0.9988 | High Risk | Yes |
| B074 | Validation | 0.9912 | High Risk | No |
| B076 | Validation | 0.7427 | High Risk | No |
| B073 | Validation | 0.3202 | Low Risk | Yes |
| B077 | Validation | 0.9970 | High Risk | No |
| B079 | Validation | 0.7598 | High Risk | No |
| B075 | Validation | 0.1514 | Low Risk | Yes |
| B078 | Validation | 0.3543 | Low Risk | No |
| B081 | Validation | 0.1446 | Low Risk | Yes |
| C247 | Validation | 0.9918 | High Risk | No |
| B127 | Validation | 0.4910 | Low Risk | Yes |
| B080 | Validation | 0.1514 | Low Risk | Yes |
| B082 | Validation | 0.6832 | High Risk | Yes |
| B128 | Validation | 0.7048 | High Risk | Yes |
| B044 | Validation | 0.9919 | High Risk | Yes |
| B125 | Validation | 0.9955 | High Risk | Yes |
| B129 | Validation | 0.9980 | High Risk | Yes |
| C256 | Validation | 0.0175 | Low Risk | Yes |
| B042 | Validation | 0.4463 | Low Risk | No |
| B045 | Validation | 0.9988 | High Risk | Yes |
| B130 | Validation | 0.1245 | Low Risk | Yes |
| B133 | Validation | 0.6214 | High Risk | Yes |
| C253 | Validation | 0.1608 | Low Risk | No |
| B060 | Validation | 0.5130 | High Risk | No |
| A345 | Validation | 0.0256 | Low Risk | Yes |
| B064 | Validation | 0.1776 | Low Risk | No |
| B059 | Validation | 0.7944 | High Risk | Yes |
| B063 | Validation | 0.5930 | High Risk | Yes |
| B065 | Validation | 0.3984 | Low Risk | Yes |
| B066 | Validation | 0.0269 | Low Risk | Yes |
| B068 | Validation | 0.9852 | High Risk | No |
| B070 | Validation | 0.9996 | High Risk | No |
| B072 | Validation | 0.0891 | Low Risk | Yes |
| B069 | Validation | 0.8449 | High Risk | No |
| C242 | Validation | 0.0570 | Low Risk | No |
| B136 | Validation | 0.9998 | High Risk | Yes |
| B137 | Validation | 0.9077 | High Risk | Yes |
| B140 | Validation | 0.0605 | Low Risk | Yes |
| B134 | Validation | 0.0191 | Low Risk | No |
| B144 | Validation | 0.9972 | High Risk | No |
| B149 | Validation | 0.9582 | High Risk | Yes |
| B152 | Validation | 0.0285 | Low Risk | Yes |
| B145 | Validation | 0.0619 | Low Risk | No |
| B147 | Validation | 0.0188 | Low Risk | Yes |
| B146 | Validation | 0.3938 | Low Risk | Yes |
| B148 | Validation | 0.0903 | Low Risk | No |
| B151 | Validation | 0.3558 | Low Risk | Yes |
| B150 | Validation | 0.3464 | Low Risk | Yes |
| B153 | Validation | 0.9364 | High Risk | Yes |
| B156 | Validation | 0.5042 | High Risk | Yes |
| A386 | Validation | 0.9811 | High Risk | Yes |
| B154 | Validation | 0.4754 | Low Risk | No |
| C263 | Validation | 0.3667 | Low Risk | No |
| B155 | Validation | 0.1070 | Low Risk | Yes |
| B083 | Validation | 0.7926 | High Risk | No |
| C254 | Validation | 0.0377 | Low Risk | Yes |
| B135 | Validation | 0.2485 | Low Risk | Yes |
| B138 | Validation | 0.0175 | Low Risk | No |
| B141 | Validation | 0.1947 | Low Risk | No |
| B043 | Validation | 0.2748 | Low Risk | Yes |
| B143 | Validation | 0.5713 | High Risk | Yes |
| B139 | Validation | 0.9569 | High Risk | Yes |
| B142 | Validation | 0.8166 | High Risk | Yes |
| B157 | Validation | 0.9930 | High Risk | No |
| B158 | Validation | 0.6104 | High Risk | Yes |
| B159 | Validation | 0.0274 | Low Risk | No |
| B162 | Validation | 0.4925 | Low Risk | No |
| B167 | Validation | 0.9635 | High Risk | No |
| B170 | Validation | 0.2312 | Low Risk | No |
| B169 | Validation | 0.0487 | Low Risk | Yes |
| B174 | Validation | 0.0876 | Low Risk | No |
| B171 | Validation | 0.2614 | Low Risk | No |
| B172 | Validation | 0.3289 | Low Risk | No |
| B175 | Validation | 0.8723 | High Risk | No |
| B165 | Validation | 0.0282 | Low Risk | Yes |
| B176 | Validation | 0.9976 | High Risk | No |
| B173 | Validation | 0.8251 | High Risk | No |
| C068 | Validation | 0.7688 | High Risk | No |
| C069 | Validation | 0.5363 | High Risk | No |
| C066 | Validation | 0.0810 | Low Risk | Yes |
| C071 | Validation | 0.2735 | Low Risk | No |
| C002 | Validation | 0.9084 | High Risk | No |
| B177 | Validation | 0.0408 | Low Risk | Yes |
| C067 | Validation | 0.5791 | High Risk | No |
| C070 | Validation | 0.9167 | High Risk | No |
| B160 | Validation | 0.1884 | Low Risk | Yes |
| B164 | Validation | 0.0319 | Low Risk | No |
| C006 | Validation | 0.2106 | Low Risk | No |
| C003 | Validation | 0.9951 | High Risk | No |
| B166 | Validation | 0.0403 | Low Risk | No |
| B168 | Validation | 0.8971 | High Risk | Yes |
| B163 | Validation | 0.9966 | High Risk | No |
| C010 | Validation | 0.9447 | High Risk | No |
| C011 | Validation | 0.0397 | Low Risk | No |
| C012 | Validation | 0.0472 | Low Risk | No |
| C015 | Validation | 0.3007 | Low Risk | No |
| C016 | Validation | 0.9278 | High Risk | No |
| C014 | Validation | 0.5624 | High Risk | No |
| C017 | Validation | 0.1205 | Low Risk | No |
| C018 | Validation | 0.0303 | Low Risk | No |
| C019 | Validation | 0.0686 | Low Risk | No |
| C020 | Validation | 0.1066 | Low Risk | No |
| C022 | Validation | 0.9996 | High Risk | No |
| C024 | Validation | 0.2787 | Low Risk | No |
| C302 | Validation | 0.5432 | High Risk | No |
| C025 | Validation | 0.9803 | High Risk | No |
| C032 | Validation | 0.4893 | Low Risk | No |
| C028 | Validation | 0.7043 | High Risk | No |
| C026 | Validation | 0.1114 | Low Risk | No |
| C035 | Validation | 0.2344 | Low Risk | No |
| C021 | Validation | 0.1242 | Low Risk | No |
| C023 | Validation | 0.9952 | High Risk | No |
| C027 | Validation | 0.1375 | Low Risk | No |
| C037 | Validation | 0.9977 | High Risk | No |
| C030 | Validation | 0.8386 | High Risk | No |
| C004 | Validation | 0.3900 | Low Risk | No |
| C008 | Validation | 0.1834 | Low Risk | No |
| C013 | Validation | 0.9415 | High Risk | No |
| C005 | Validation | 0.7151 | High Risk | No |
| C007 | Validation | 0.9896 | High Risk | No |
| C009 | Validation | 0.4655 | Low Risk | No |
| C029 | Validation | 0.0361 | Low Risk | No |
| C031 | Validation | 0.9574 | High Risk | No |
| C039 | Validation | 0.9546 | High Risk | No |
| C038 | Validation | 0.2350 | Low Risk | No |
| C041 | Validation | 0.9355 | High Risk | No |
| C036 | Validation | 0.5410 | High Risk | No |
| C040 | Validation | 0.0387 | Low Risk | No |
| C042 | Validation | 0.3232 | Low Risk | No |
| C307 | Validation | 0.2258 | Low Risk | No |
| C033 | Validation | 0.9769 | High Risk | No |
| C044 | Validation | 0.0861 | Low Risk | No |
| B131 | Validation | 0.4148 | Low Risk | No |
| C001 | Validation | 0.8941 | High Risk | No |
| C034 | Validation | 0.0610 | Low Risk | No |
| C363 | Validation | 0.6943 | High Risk | Yes |
| C357 | Validation | 0.9028 | High Risk | No |
| B206 | Validation | 0.4480 | Low Risk | No |
| B207 | Validation | 0.9784 | High Risk | No |
| C631 | Validation | 0.2967 | Low Risk | Yes |
| B203 | Validation | 0.0588 | Low Risk | No |
| B209 | Validation | 0.3127 | Low Risk | No |
| B208 | Validation | 0.5090 | High Risk | No |
| B210 | Validation | 0.9966 | High Risk | No |
| B211 | Validation | 0.9591 | High Risk | No |
| B212 | Validation | 0.4641 | Low Risk | No |
| B213 | Validation | 0.1401 | Low Risk | No |
| B215 | Validation | 0.5955 | High Risk | No |
| B217 | Validation | 0.9053 | High Risk | No |
| B220 | Validation | 0.7923 | High Risk | No |
| B221 | Validation | 0.6898 | High Risk | No |
| B222 | Validation | 0.3758 | Low Risk | No |
| B219 | Validation | 0.1259 | Low Risk | No |
| B223 | Validation | 0.1179 | Low Risk | No |
| B216 | Validation | 0.6620 | High Risk | No |
| C672 | Validation | 0.0260 | Low Risk | Yes |
| B218 | Validation | 0.9983 | High Risk | No |
| B214 | Validation | 0.8199 | High Risk | No |
| C694 | Validation | 0.1127 | Low Risk | Yes |
| C058 | Validation | 0.2179 | Low Risk | No |
| C377 | Validation | 0.0687 | Low Risk | No |
| A390 | Validation | 0.9993 | High Risk | No |
| C374 | Validation | 0.0254 | Low Risk | No |
| C381 | Validation | 0.9445 | High Risk | No |
| C385 | Validation | 0.1770 | Low Risk | No |
| C391 | Validation | 0.1796 | Low Risk | No |
| C392 | Validation | 0.0290 | Low Risk | No |
| C396 | Validation | 0.0958 | Low Risk | No |
| C400 | Validation | 0.1955 | Low Risk | No |
| C409 | Validation | 0.8060 | High Risk | No |
| C418 | Validation | 0.0530 | Low Risk | No |
| C393 | Validation | 0.9965 | High Risk | No |
| C433 | Validation | 0.0650 | Low Risk | No |
| C436 | Validation | 0.6070 | High Risk | No |
| C444 | Validation | 0.1839 | Low Risk | No |
| C434 | Validation | 0.1265 | Low Risk | No |
| C456 | Validation | 0.3545 | Low Risk | No |
| C461 | Validation | 0.1328 | Low Risk | No |
| C460 | Validation | 0.1453 | Low Risk | No |
| C463 | Validation | 0.5041 | High Risk | No |
| C467 | Validation | 0.0348 | Low Risk | No |
| C424 | Validation | 0.3093 | Low Risk | No |
| C428 | Validation | 0.8879 | High Risk | Yes |
| C499 | Validation | 0.0831 | Low Risk | No |
| C495 | Validation | 0.1938 | Low Risk | No |
| C498 | Validation | 0.3303 | Low Risk | No |
| C503 | Validation | 0.0388 | Low Risk | No |
| C511 | Validation | 0.9819 | High Risk | No |
| C501 | Validation | 0.9949 | High Risk | No |
| C475 | Validation | 0.4351 | Low Risk | No |
| C488 | Validation | 0.0987 | Low Risk | No |
| C520 | Validation | 0.1097 | Low Risk | No |
| C507 | Validation | 0.9977 | High Risk | No |
| C509 | Validation | 0.1016 | Low Risk | No |
| C504 | Validation | 0.1222 | Low Risk | No |
| C515 | Validation | 0.7645 | High Risk | No |
| C526 | Validation | 0.1797 | Low Risk | No |
| C470 | Validation | 0.9919 | High Risk | No |
| C479 | Validation | 0.0285 | Low Risk | Yes |
| C474 | Validation | 0.5704 | High Risk | No |
| C522 | Validation | 0.0553 | Low Risk | No |
| C532 | Validation | 0.3156 | Low Risk | No |
| C528 | Validation | 0.6760 | High Risk | No |
| C549 | Validation | 0.2065 | Low Risk | No |
| C556 | Validation | 0.9051 | High Risk | No |
| B178 | Validation | 0.4240 | Low Risk | No |
| C513 | Validation | 0.8760 | High Risk | No |
| B182 | Validation | 0.5688 | High Risk | No |
| B184 | Validation | 0.9742 | High Risk | No |
| C530 | Validation | 0.5172 | High Risk | No |
| C540 | Validation | 0.8357 | High Risk | No |
| C543 | Validation | 0.2696 | Low Risk | No |
| B183 | Validation | 0.0608 | Low Risk | No |
| B185 | Validation | 0.0891 | Low Risk | No |
| B186 | Validation | 0.7309 | High Risk | No |
| B187 | Validation | 0.7044 | High Risk | No |
| B188 | Validation | 0.4949 | Low Risk | No |
| B189 | Validation | 0.4421 | Low Risk | No |
| B193 | Validation | 0.0740 | Low Risk | No |
| B192 | Validation | 0.4915 | Low Risk | No |
| B194 | Validation | 0.9542 | High Risk | No |
| B179 | Validation | 0.9996 | High Risk | No |
| B195 | Validation | 0.9872 | High Risk | No |
| B196 | Validation | 0.9893 | High Risk | No |
| B204 | Validation | 0.0442 | Low Risk | No |
| C118 | Validation | 0.3041 | Low Risk | No |
| B191 | Validation | 0.9804 | High Risk | No |
| B200 | Validation | 0.9107 | High Risk | No |
| B201 | Validation | 0.4933 | Low Risk | No |
| B202 | Validation | 0.0529 | Low Risk | No |
| B197 | Validation | 0.9908 | High Risk | No |
| C448 | Validation | 0.8102 | High Risk | No |
| B199 | Validation | 0.8145 | High Risk | No |
| B116 | Validation | 0.0275 | Low Risk | No |
| C833 | Validation | 0.9886 | High Risk | No |
| C757 | Validation | 0.0861 | Low Risk | No |
| C866 | Validation | 0.9973 | High Risk | Yes |
| C872 | Validation | 0.5868 | High Risk | Yes |
| B126 | Validation | 0.0502 | Low Risk | No |
| C795 | Validation | 0.1725 | Low Risk | Yes |
| D405 | Validation | 0.8758 | High Risk | Yes |
| D406 | Validation | 0.0665 | Low Risk | Yes |
| D408 | Validation | 0.8909 | High Risk | Yes |
| D407 | Validation | 0.6425 | High Risk | Yes |
| C960 | Validation | 0.3014 | Low Risk | Yes |
| C962 | Validation | 0.9921 | High Risk | Yes |
| C963 | Validation | 0.2435 | Low Risk | Yes |
| C964 | Validation | 0.1290 | Low Risk | Yes |
| C965 | Validation | 0.9488 | High Risk | Yes |
| C966 | Validation | 0.3589 | Low Risk | No |
| B132 | Validation | 0.8929 | High Risk | No |
| D413 | Validation | 0.6411 | High Risk | Yes |
| D411 | Validation | 0.7786 | High Risk | Yes |
| D412 | Validation | 0.9124 | High Risk | Yes |
